# Scientific misconduct: our first (known) case

**DOI:** 10.1590/S1679-45082014ED3296

**Published:** 2014

**Authors:** Jacyr Pasternak

**Affiliations:** 1 Hospital Israelita Albert Einstein, São Paulo, SP, Brazil.

**einstein** registered (in the last issue) its first retraction, due to a case of duplicate publication: “Neuromuscular electrical stimulation in critically ill patients in the intensive care unit: a systematic review”, by Lucas Lima Ferreira, Luiz Carlos Marques Vanderlei and Vitor Engrácia Valenti. Our journal, like all peer-reviewed and indexed journals, asks authors to state unequivocally in their submission letter that the paper has not already been submitted to another journal. This letter is signed by all the authors, so we take this affirmation as a fact. In this case this fact was fiction…

Scientific misconduct has many faces: duplicate publication is perhaps the easiest to discover. It was more common in the past when some so-called scientists, pressured for publications (“publish or perish”), sought little-known journals in non-English languages and tried to submit papers to several journals at once. As indexing and searching systems improved, this practice became more and more difficult. A comprehensive analysis of retracted articles in the medical literature between 2004 and 2013^(1)^ showed an increase in numbers of retracted articles in recent years. Most of these retractions are original articles followed by case reports.

A recent paper by Lins and Carvalho^(2)^ analyzed scientific misconduct in Brazil. They found a clear increase in both published articles in the medical literature and cases of scientific misconduct, including irreproducible results, “scientific salami slicing” (one article fragmented into 10 or more papers) and duplicate publications. In Lins and Carvalho’s opinion, the increased number of Brazilian scientific productions in medical literature was not accompanied by an increase in quality of articles – just the opposite. The authors discuss the focus of Brazilian institutional review boards in patient safety, within institutions themselves and the Brazilian National Review Board. Neither group performs a systematic surveillance for research integrity, and no specific offices exist to investigate and deal with scientific misconduct. Editors’ efforts can at least decrease duplicate publications: Korean authors noted a duplicate publication rate as high as 5.9% of all articles published in Korea in 2004, 6.0% in 2005, and 7.2% in 2006. To reverse this increase in duplicate publication, a comprehensive database of all Korean medical articles published by Korean authors in indexed and non-indexed journals was created. The campaign to end duplicate publication achieved successful results: in 2009 only 1.2% of all articles published were duplicates.^(3)^

A recent paper on manuscript submissions reported that recently most journals have noted a clear increase in ethically dubious articles, and this means a significant surcharge to editors. Such papers are received, reviewed and sometimes – what is worse – published. Editors are not supposed to be detectives: when people declare in writing that their paper is original, produced with real data, not invented ones; that experiments were done as written; that results were not tortured to gain statistical significance; that the complete work was submitted; and that the paper was not previously submitted to other journal, editors do believe.^(4)^ How should we deal with duplicate submissions? A recent letter to the editor in an Iranian journal gives excellent suggestions for this question that our journal will follow.^(5)^

– editors should not allow any author of a duplicate submission to ever publish again in their journal;

– both journals involved in duplicate submissions should communicate that fact to indexing and other databases, again including the names of all authors (or we shall call them “duplicators?”);

– if the authors are from a university or are receiving public financing, the responsible authorities should be informed.

## References

[1] Singh HP, Mahendra A, Yadava B, Singh H, Arora N, Arora M (2014). A comprehensive analysis of articles reatracted between 2004 and 2013 from the biomedical literature – a call for reform. J Tradit Compl Med.

[2] Lins L, Carvalho FM (2014). Scientific integrity in Brazil. J Bioeth Inq.

[3] Kim SY, Bae CW, Hahm CK, Cho HM (2014). Duplicate publication rate decline in Korean medical Journals. J Korean Med Sci.

[4] Murphy SP, Bulman C, Shariati B, Hausmann L, ISN Publications Committee (2014). Submiting a manuscript for peer review: integrity, integrity, integrity. J Neurochem.

[5] Yahyavi ST (2014). Facing to duplicate submission as scientific misconduct. Iran J Psychiatry Behav.

