# Supplementary material for: Scientific misconduct: our first (known) case
Source: Einstein (Sao Paulo). 2014 Oct-Dec;12(4):7–8. doi: 10.1590/S1679-45082014ED3296 (PMC4879902; doi:10.1590/S1679-45082014ED3296)
Supplement: Translation

# Translation: Má conduta científica: nosso primeiro caso (reconhecido)

**Authors:** Jacyr Pasternak

**Affiliations:** 1 Hospital Israelita Albert Einstein, São Paulo, SP, Brasil.

A **einstein** registrou, no fascículo anterior, a primeira retratação em razão de um caso de publicação duplicada: “Estimulação elétrica neuromuscular em pacientes graves em unidade de terapia intensiva: revisão sistemática”, por Lucas Lima Ferreira, Luiz Carlos Marques Vanderlei e Vitor Engrácia Valenti. Nosso periódico, como todo periódico indexado e revisado pelos pares, solicita aos autores declaração inequívoca em suas cartas de submissão assegurando que o artigo não foi anteriormente submetido a outro periódico. Tal carta é assinada por todos os autores, portanto, essa afirmação é considerada um fato. Neste caso, o fato era fictício...

A má conduta científica tem muitas faces: a publicação duplicada, talvez, seja uma das mais fáceis de descobrir. Essa prática era mais comum no passado, quando alguns “cientistas”, pressionados a publicar (“publique ou você dança”), procuravam periódicos pouco conhecidos, que não publicavam em língua inglesa, e tentavam submeter seus manuscritos a diversos deles ao mesmo tempo. Com a melhora dos sistemas de busca e de indexação, essa prática tornou-se mais e mais difícil de realizar. Uma análise de artigos retratados na literatura médica entre 2004 e 2013^(1)^ mostrou aumento no número de artigos retratados nos últimos anos. A maioria das retratações foi de artigos originais, seguidos de relatos de casos.

O artigo recente de Lins e Carvalho ^(2)^ analisou a má conduta científica no Brasil. Esses autores encontraram um claro aumento na publicação de artigos em literatura médica e de casos de má conduta científica, incluindo resultados irreproduzíveis, “ciência salame” (um artigo fragmentado em 10 ou mais artigos) e publicações duplicadas. Na opinião de Lins e Carvalho, o aumento no número de produções científicas brasileiras na literatura médica não foi acompanhado da melhora na qualidade dos artigos – mas, sim, do oposto. Os autores discutem o foco dos Conselhos de Ética em Pesquisa brasileiros, nas próprias instituições e na Comissão Nacional de Ética em Pesquisa (CONEP). Nenhum destes realiza vigilância sistemática para integridade em pesquisa, e inexistem órgãos específicos para investigar e lidar com a má conduta científica. Os esforços dos editores podem, pelo menos, diminuir a publicação duplicada: autores coreanos notaram altas taxas de publicações duplicadas de 5,9% em relação a todos os artigos publicados na Coreia em 2004, de 6,0% em 2005 e de 7,2% em 2006. Para reverter esse aumento de publicações duplicadas, foi elaborada uma base de dados, incluindo todos os manuscritos coreanos em medicina publicados por autores coreanos em periódicos indexados e não indexados. A campanha para finalizar a publicação duplicada alcançou sucesso: em 2009, apenas 1,2% de todos os artigos publicados eram duplicidades.^(3)^

Um artigo recente sobre submissão de artigos relatou que a maioria dos periódicos tem notado um claro aumento de artigos eticamente duvidosos, representando, portanto, uma sobrecarga significante aos editores. Tais artigos são recebidos, revisados e algumas vezes – o que é pior – publicados. Os editores não são detetives: quando os autores declaram por escrito que seu artigo é original, produzido com dados reais, não inventados; que os experimentos foram feitos como descrito; que os resultados não foram distorcidos para obter significância estatística; que o trabalho completo foi submetido; e que o artigo não foi previamente submetido a outra publicação, os editores acreditam em tais afirmações.^(4)^ Como devemos lidar com submissões duplicadas? Uma carta ao editor publicada recentemente em um periódico iraniano dá sugestões excelentes para essa questão, as quais serão seguidas por nosso periódico.^(5)^

– os editores não devem permitir qualquer autor de submissão duplicada que publique novamente em seus periódicos;

– os periódicos envolvidos em submissões duplicadas devem comunicar o fato para base de dados indexadoras e outras bases, incluindo também os nomes de todos os autores (ou devemos chamá-los de “duplicadores?”);

– se os autores são de universidade ou estão recebendo financiamento público, as autoridades responsáveis devem ser informadas.
